# Hybrid Nonlinear Metasurface Refractive Lens

**DOI:** 10.1021/acs.nanolett.5c00178

**Published:** 2025-04-03

**Authors:** Sharon Karepov, Costantino De Angelis, Tal Ellenbogen

**Affiliations:** † Department of Physical Electronics, Faculty of Engineering, 26745 Tel-Aviv University, Tel-Aviv 6997801, Israel; ‡ Light Matter Interaction Center, Tel Aviv University, Tel Aviv 6997801, Israel; § Department of Information Engineering, 9297 University of Brescia, Via Branze 38, 25123 Brescia, Italy

**Keywords:** Metasurface applications, nonlinear plasmonic metasurfaces, metasurfaces on curved substrates, metasurfaces in thin films, metasurface transferring

## Abstract

Nonlinear metasurfaces have been extensively studied, offering new capabilities to generate and control light. Usually, nonlinear metasurfaces are fabricated on planar substrates where the entire functionality is encoded in the metasurface design. Here, we study a new route to nonlinear metasurface-based optics by coating linear refractive elements with conformable nonlinear metasurface-skin, resulting in hybrid nonlinear refractive elements. We specifically demonstrate a hybrid nonlinear meta-lens composed of a plano-convex linear refractive lens coated with a 400 nm thick nonlinear metasurface membrane. We show that the resulting hybrid element produces light at the second harmonic frequency and focuses it according to the hybrid linear–nonlinear optical functionality. We study the spectral and polarization responses of the hybrid element and demonstrate its ability to generate an image at the second harmonic wavelength. We believe that this demonstration opens the door to a new family of hybrid nonlinear refractive elements for controlling light in new applications.

## Graphical abstract

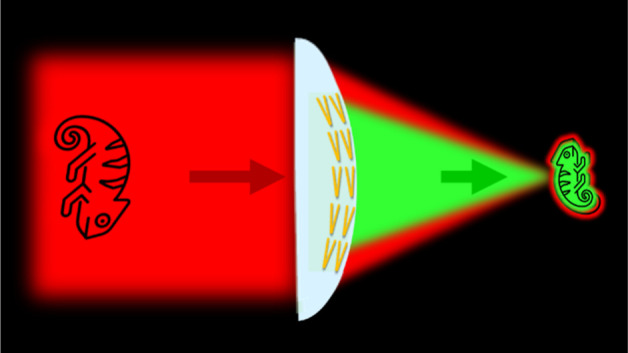

Optical metasurfaces are ultrathin nanoengineered planar films composed of subwavelength building blocks, commonly known as meta-atoms. Their interaction with light is determined by the building blocks’ material composition, shape, orientation, dimensions, and spatial arrangement.^1,2^ With the proper design, these elements can provide spatial, spectral, amplitude, polarization, and phase control.^3^ Therefore, metasurface applications are numerous and include polarization and spectrally multiplexed holography,^4,5^ beam shaping,^6^ emission control,^7^ frequency conversion,^8^ etc.

In addition to applications in linear optics, by constructing the metasurfaces from nonlinear optical materials, or by breaking the inversion symmetry through design, a nonzero second order susceptibility can be obtained, which supports a second order nonlinear response.^9^ Such nonlinear metasurfaces have gained increasing interest, thanks to the ability to fully control their tensorial properties^10^ and to engineer their operational band,^11–13^ as well as controlling the output signal polarization^14,15^ and longitudinal and transverse modes.^16,17^

The most common fabrication processes of metasurfaces involves techniques, such as electron beam lithography, which requires rigid substrates, like silicon or glass,^1,18,19^ and, usually, results in planar metasurfaces on the used substrate. However, recently, there have been developments of fabrication schemes for obtaining flexible and transferable metasurfaces, which paved the way for completely new applications.^20–22^ For example, deforming flexible metasurfaces can produce a frequency-tunable optical response used for cloaking,^23,24^ for demonstrations of active color pixels^25,26^ and sensing devices.^22^ In addition, metasurface membranes were transferred to nonplanar glasses to project off-axis holograms^27^ and to contact lenses, which can functionalize them with new abilities.^28^

All of these exciting applications emphasize the strength of transferable metasurface platforms for integration in various optical systems and for obtaining new functionalities. Here, we use it to explore a new concept: functionalization of linear refractive optical elements with nonlinear metasurface films. This lays the foundation to turn conventional optical components into nonlinear optical elements for various applications, such as for second harmonic (SH) microscopy,^29,30^ optical sensing,^31^ and upconversion imaging.^32,33^ To this end, we report the first, to the best of our knowledge, hybrid nonlinear metasurface refracting lens (HNML). The HNML is composed of a nonlinear metasurface transferred to an off-the-shelf lens. The metasurface grants the lens new functionality, i.e., generating SH light, while preserving the lens refraction property. That is, the rays of both the SH light and the incident fundamental frequency (FF) are effectively refracted in the same way. We report on the spectral response of the element as well as demonstrate its performance as a nonlinear lens that focuses the FF and SH light and can be used for nonlinear imaging at the SH.

Our nonlinear metasurface comprises a collection of non-centrosymmetric gold V-shaped nanoparticles composed of two equally long perpendicular arms. We chose this structure for our meta-atoms since they are known to have an artificial quadratic nonlinearity.^11,34^ Several studies have shown that the artificial quadratic nonlinearity originates from the break in the local symmetry and is enhanced by the resonant response of the metasurface.^34–38^

A scanning electron microscope (SEM) image and an illustration of the metasurface after the first fabrication step, which is elaborated in section S1 of the Supporting Information, are shown in Figure 1(a). The Vs’ thickness is 40 nm, their arm length and width are 135 and 50 nm, respectively, and the overall metasurface size is 1 × 1 mm^2^. These meta-atoms are arranged in a square lattice with a periodicity of 260 nm, significantly smaller than the SH wavelengths, in the range of 520–780 nm. Hence diffraction and nonlocal resonant effects are negligible.

**Figure 1:**
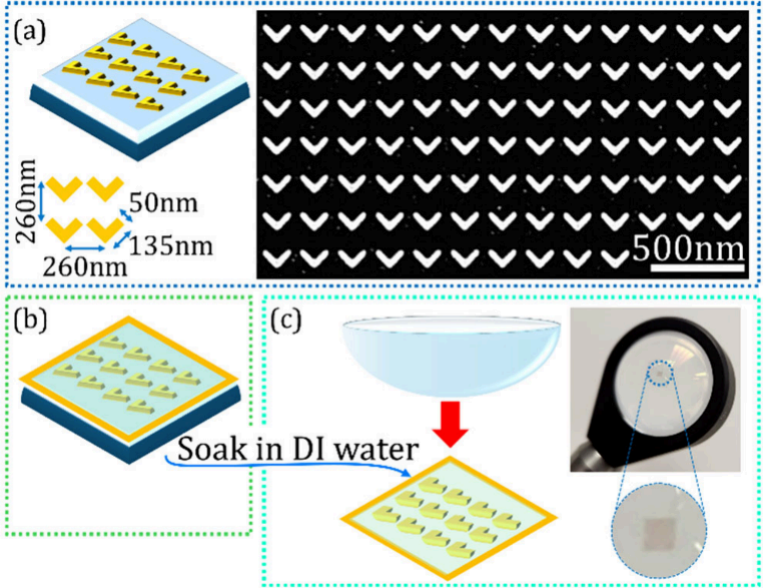
HNML fabrication steps. (a) V-shaped meta-atoms on an indium-tin-oxide coated glass illustration, left; SEM image of the meta-atoms, right. (b) Element after PMMA spin coat and Kapton tape frame. (c) Free standing metasurface membrane being attached to the goal substrate, left; the final HNML element, top right; zoom in on the region of the metasurface, bottom right.

To prepare the nonlinear metasurface skin designated to coat and functionalize the refractive lens, we coated the metasurface with a thin poly­(methyl methacrylate) (PMMA) layer, which preserved the metasurface arrangement while it was later relocated. To prevent membrane deformations, a Kapton frame was taped around the metasurface area over the PMMA, as illustrated in Figure 1(b). Then, we soaked the element in deionized (DI) water and released the PMMA-metasurface film from the ITO-coated glass. In general, the resulting metasurface-PMMA film can be utilized as a free-standing thin membrane, where it can also be attached to a variety of other substrates, thanks to the PMMA inherent adhesiveness and fabric-like ability to deform.^28^ Other than that, PMMA has four more properties that are instrumental for the concept of transferring metasurfaces. First, the low viscosity of PMMA enables it to flow between the meta-atoms, during the initial drop cast, and encapsulate them. In addition, by immersing the element in water, capillary forces rise and can separate the PMMA layer from a hydrophilic substrate, like glass.^39,40^ Moreover, it is possible to spin coat ultrathin layers of PMMA, a feature that can facilitate the integration of a metasurface–PMMA membrane into a large diversity of applications. Finally, PMMA is transparent across the visible and near-infrared spectrum,^41^ a property necessary to achieve a strong signal throughout the entire optical range we are interested in.

Once the membrane was free-standing, we attached it to the convex side of an off-the-shelf refractive lens, with a linear focal length marked by *f*_L‑HNML_, which, according to the manufacturer’s specifications, is 25.4 mm. This step and the resulting HNML are presented in Figure 1(c), left and right, respectively.

To investigate the optical performance of the HNML, we used a measurement setup in a transmission configuration as depicted in Figure 2, where the design is detailed in the Experimental Methods section. The linear properties of the HNML were studied by illuminating it with a supercontinuum laser and recording the transmitted light. For the nonlinear measurements, we used a tunable femtosecond optical parametric oscillator pumped by a titanium sapphire laser (pulse width ∼ 140 fs, repetition rate 80 MHz) as the FF source with a wavelength span in the range of 1040–1580 nm. The polarization and power of the FF were controlled by a broadband half-waveplate (λ/2) and a wire grid polarizer (P). A lens, denoted by L1, focused the light that traveled toward the HNML. The focal length of L1 and the distance between L1 and the HNML are denoted by *f*_L1_ and *d*_1_, respectively. From the HNML the light propagated to a long working distance (WD) microscope objective lens (MO) and to its compatible tube lens (TL), which defined the image plane. To analyze the image of the generated SH beam across different planes of the optical axis, we define the spacing between the HNML and the WD plane as *d*_2_. Next, two identical lenses, L2 and L3, were set in a 4f configuration to project the light onto our detectors.

**Figure 2:**
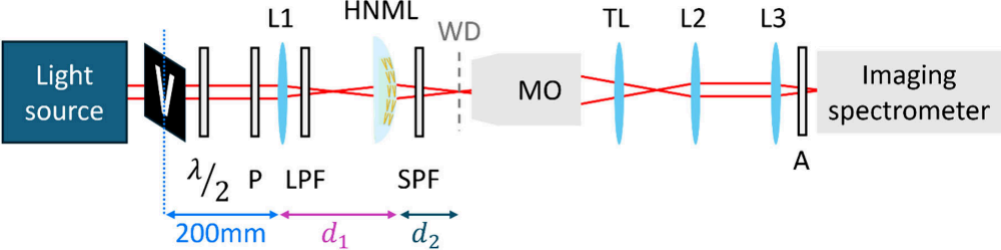
Measurement setup. λ/2, half-waveplate; P, linear polarizer; L1, L2, L3, 200 mm focal length lenses; LPF and SPF, long and short pass filters, respectively, used for nonlinear measurements; MO, microscope objective lens with a working distance denoted by WD; TL, tube lens; A, analyzer. The parameters *d*_1_ and *d*_2_ correspond to the distances between L1 and the HNML and between the HNML and the WD plane of the MO lens. Three slightly different configurations were utilized: (i) Light was focused on the HNML by setting *d*_1_ = *f*_L1_, *d*_2_ = 0 mm. (ii) The HNML imaged the focused spot created by lens L1. Here *f*_L1_ < *d*_1_ and *d*_2_ was modified in order to scan the beam across the optical axis, including the image plane. (iii) L1 and the HNML were set in imaging configuration, in which the object, an opaque rectangle with a V-shaped slit, was placed at the front focal plane of L1. In this case, *d*_1_ = *f*_L1_ + *f*_L‑HNML_ and *d*_2_ = *f*_L‑HNML_.

During the nonlinear measurements, long and short pass filters, indicated by LPF and SPF, respectively, were incorporated into the setup with the intention to block SH light that may not originate from the proposed element. In addition, for SH polarization resolved analysis, a broadband polarizer (A) was added before the imaging spectrometer. We used three slightly different configurations of the setup: (i) SH was generated when the FF light was focused on the HNML and collected right after it by setting *d*_1_ to *f*_L1_ and *d*_2_ to 0 mm; (ii) the HNML imaged the focused spot created by lens L1. Here *f*_L1_ < *d*_1_ and *d*_2_ was modified in order to scan the beam across the optical axis, including the image plane. By scanning *d*_2_, one may observe the SH image formation; (iii) L1 and the HNML were placed in an imaging configuration, in which the object, depicted by an opaque rectangle with the V-shaped slit in Figure 2, was placed at the front focal plane of lens L1. To perceive an image, we set the values *d*_1_ = *f*_L1_ + *f*_L‑HNML_ and *d*_2_ = *f*_L‑HNML_. As will be discussed, with every setup configuration we explored a different characteristic of the HNML.

## Results

The linear response of the metasurface was characterized by measuring the polarized spectral transmission, as displayed in Figure 3(a). The polarization orientation with respect to the V-shaped meta-atom can be seen in the illustration of Figure 3(a), right. More specifically, in this figure orange and magenta indicate horizontally (H-pol) and vertically (V-pol) polarized illumination, correspondingly. The solid lines show the experimental measurements, and the dashed lines show the transmission obtained from a finite difference time domain (FDTD) simulation conducted in Ansys Lumerical. A relatively good agreement is obtained between the measurements and the simulations over the entire spectral region, specifically indicating the measured resonances. However, there are also some noticeable differences between the measured and simulated transmission curves. We believe that these deviations originate from the difference between the simulation and the measurement conditions. As opposed to the plane wave illumination defined in our simulations, as elaborated in section S2 of the Supporting Information, in our measurement setup, the HNML was illuminated by a spherical wave, since the light was focused by lens L1, prior to its arrival at our HNML. Therefore, effectively, the metasurface is illuminated by multiangle wavevectors, causing the observed cross-talk in the spectral response. It can be seen that the resonance of horizontally polarized excitation is obtained at a wavelength of 1085 nm and it is red-shifted in comparison to its orthogonal polarized excitation, in which the maximal extinction occurs at the wavelength 715 nm. This shift is in agreement with previous studies.^9^

**Figure 3:**
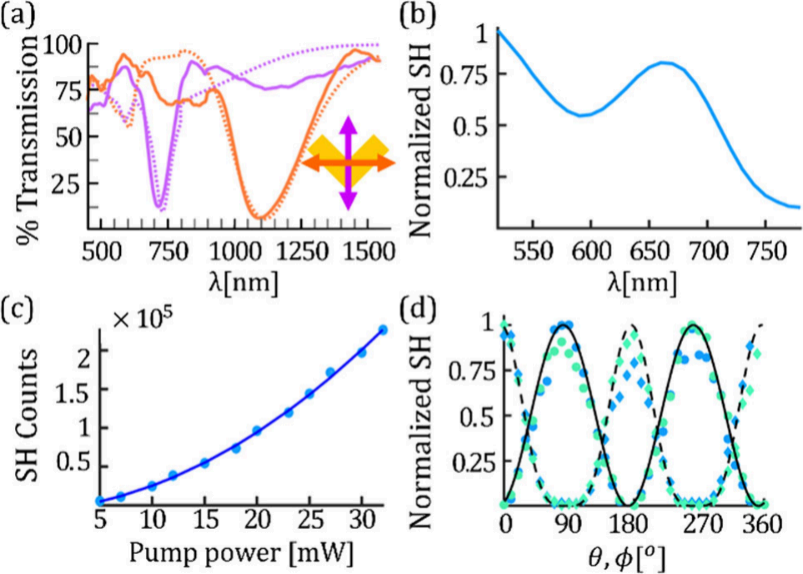
Optical characterization of the HNML. (a) Spectral transmission of H-pol and V-pol light in orange and magenta curves, respectively, as seen in the meta-atomillustration on the bottom right. Solid and dashed curves describe measurements and simulations results, respectively. (b) SH counts vs. SH wavelength. (c) SH counts vs. FF pump power. (d) Dependence of the recorded SH counts in the pump polarization angle, θ, and in the analyzer angle, ϕ, both with a constant pump power. The first is depicted as scattered diamonds and the latter as dots. Solid and dashed black curves correspond to cos^2^(ϕ) and cos^4^(θ). Blue and cyan data present measurements recorded with the setup configurations described as (i) and (ii) with *d*_2_ = 40 mm, correspondingly.

The characterization of the nonlinear optical response was done by recording the SH signal for different pump wavelengths in the range of 1040–1580 nm using a constant FF power of 30 mW. During this study, the pump power did not exceed 50 mW, in order to avoid burning the meta-skin. Thermal damage is important to take into account since the presented HNML is composed of plasmonic nanoparticles, which are subjected to significant Ohmic losses.^1,42^ This is an issue that can be greatly improved by using nonlinear dielectric metasurfaces.^43,44^

We concentrated on SH generated by an H-pol excitation, which generates V-pol SH light,^11,34,45^ in a similar manner to SH from split ring resonators.^37^ Figure 3(b) shows the obtained SH counts as a function of the generated SH wavelength. We obtain the strongest SH at 520 nm in spectral close proximity to the resonant interaction with the H-Pol FF wave and an SH peak at ∼660 nm due to improved field overlap with the V-Pol SH resonance.

The expected quadratic dependence of the SH power on the FF power^46^ is displayed in Figure 3(c). Here, the HNML was excited by a 1320 nm pump wavelength. Generally, the conversion efficiency of plasmonic metasurfaces is of the order of 10^–11^–10^–9^ for similar excitation characteristics.^11,16^ The conversion efficiency can be enhanced by using higher intensities, however, in the case of plasmonic metasurfaces, as used in our work, the damage threshold needs to be avoided. In order to improve the conversion efficiency and increase the damage thresholds, nonlinear dielectric metasurfaces can be used.^47,48^

In addition, we measured the nonlinear polarization response of the SH, either by fixing the FF polarizer to H-pol and rotating the SH analyzer or by fixing the SH analyzer to V-pol and rotating the FF polarizer. The results are presented in Figure 3(d), for illumination of the HNML with a 30 mW excitation at a wavelength of 1320 nm in configurations (i) and (ii) in blue and cyan, respectively. The FF polarizer angle is denoted by θ, and ϕ is the angle of the SH analyzer. The dependence on the SH analyzer shows cos^2^(ϕ) function, according to Malus law,^46^ and the dependence of the SH on the pump polarizer shows cos^4^(θ), due to the quadratic nonlinear interaction.

We now turn to examine the hybrid performance of the element. While the nonlinear metasurface functionalizes the refractive element and turns it into a nonlinear optical element, the refractive properties of the hybrid element are preserved. For each angle of incidence of the FF wave, *θ*_*i*_(ω), the angle of refraction of the SH wave can be calculated by conservation of the momentum parallel to the interface 2*k*_ω_^∥^ = *k*_2ω_^∥^. This relation leads to a nonlinear Snell’s law *n*_*i*_(ω) sin *θ*_*i*_(ω) = *n*_*t*_(2ω) sin* θ*_*t*_(2ω), where *θ*_*t*_(2ω) is the transmitted angle of the SH and *n*_*i*_ and *n*_*t*_ are refractive indices of the incident and transmitted media, respectively. For the case of transmission from glass to air, the nondispersive characteristics of air lead to *θ*_*t*_(2ω) = *θ*_*t*_(ω). Therefore, the hybrid lens not only generates SH light but also refracts it equivalently to the refraction of the incident FF. This phenomenon can be considered as effective refraction, since the light at the SH frequency does not propagate in the lens glass directly but inherits the refraction of the FF wave through the conversion process.

The imaging condition of a thin lens, with a focal length represented by *f*, is given by 1/*u* + 1/*v* = 1/*f*,^46^ where the distances between the lens and the planes of the object and the image are denoted by *u* and *v*, respectively. Therefore, by setting *d*_1_ to ∼266 mm in configuration (ii) and gradually increasing *d*_2_ within the range of 0–50 mm, one may witness how the SH converges to a focused spot with the corresponding increase in its intensity.

To measure SH focusing, we increased *d*_2_ while recording images of the SH spot. In every image, we extracted the beam spot size from the number of pixels with an SH count that was equal or larger than 13.5% of the maximal SH counts value, in accordance with a Gaussian beam approximation.^46^ Then, for every *d*_2_, the spot size was normalized, with respect to that at the focal plane. These results can be seen in Figure 4(a) as a scattered red rhombus, with corresponding images beneath the chart. The observed astigmatism is due to a 2.5 mm deviation of the metasurface from the center of the lens, as can be seen in Figure 1(c). It can be seen how the spot size of the SH beam narrows until the SH is focused at *d*_2_ = 40 mm, followed by the divergence of the beam. In order to validate these findings, we calculated the propagation of a Gaussian beam through L1 and a 25.4 mm focal length lens with the same physical parameters as in our experimental setup using the ray transfer matrix analysis.^46^ The resulting spot size vs. *d*_2_ is presented as a solid blue line in Figure 4(a), showing good agreement with our measured focusing. Figure 4(b) presents the maximal detected counts in every image. As *d*_2_ becomes larger, the SH maximal count increases until reaching the focal plane, where afterward, due to the beam divergence, the SH intensity decreases.

**Figure 4:**
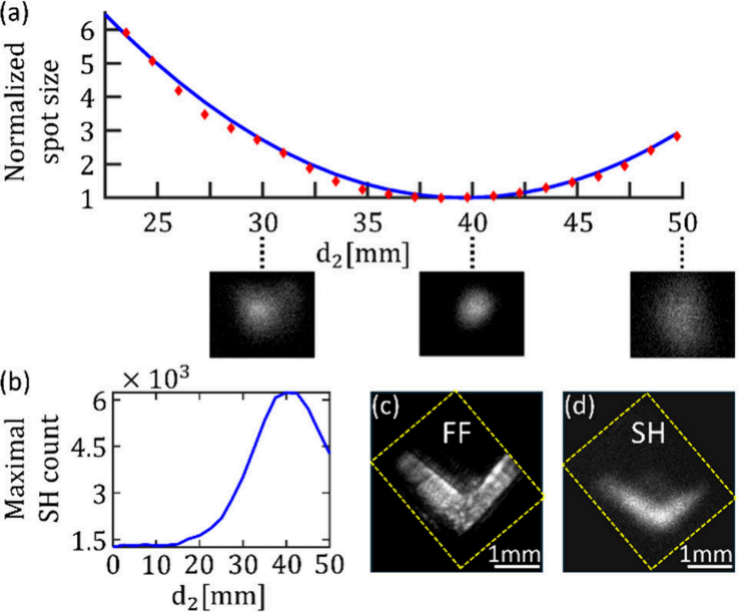
(a, b) Characterization of the generated SH spot with respect to the light collection plane, *d*_2_. Measurements were recorded with the (ii) setup configuration. (a) Normalized SH spot size vs. *d*_2_. The solid line displays the result of a ray transfer matrix analysis, and the red rhombs show the measured size of the SH beam. The measurements and simulation results were normalized with their corresponding spot size at *d*_2_ = 40 mm. Below the chart there are three images of the spot, recorded at different *d*_2_’s across the optical axis. (b) Maximal SH counts vs. *d*_2_. (c, d) Imaging using the HNML. The captured images were slightly rotated, for display purposes, and they are presented with a dashed yellow frame and a black background behind them. (c) Linear image of a V-shaped slit illuminated with a wavelength of 1100 nm. (d) SH image of the V-shaped slit.

We then performed a nonlinear imaging experiment in which the object is illuminated by the FF beam and the image is encoded on the FF and SH beams. For this purpose, the experimental setup was set in configuration (iii), in which L1 and the HNML constituted an imaging system, since *d*_1_ was equal to the sum of L1 and our HNML focal lengths and *d*_2_ was adjusted to the HNML focal length, which is *f*_L‑HNML_. With this layout, we imaged an opaque glass with a V-shaped slit that was placed at the front focal plane of lens L1, as shown in Figure 2.

An image of the slit, illuminated with a wavelength of 1100 nm, is displayed in Figure 4(c), and the corresponding SH image is presented in Figure 4(d), where the pump was H-pol and the excitation was performed with a wavelength of 1320 nm. The captured images were slightly rotated for display purposes, and they are presented with a dashed yellow frame and a black background behind them. It can be seen how the V-shaped slit was imaged both with the near-infrared and with the generated SH beam, demonstrating the nonlinear imaging capabilities of the HNML. The difference in imaging quality between the linear and SH images can be attributed to the lower intensity of the generated SH at the image plane as opposed to a condition where the SH is focused. The lower signal-to-noise ratio deteriorates the image quality since high spatial frequencies become undetectable. This issue can be improved by increasing the conversion efficiency, for example, with the use of nonlinear dielectric metasurface skins, to receive a stronger SH signal and, thus, a sharper image.^47,48^

Since the presented element is, essentially, the hybridization of two elements, the metasurface and the refractive lens, the advantages and limitations of both determine the total optical response. As stated previously, the generated SH light experiences effective refraction that corresponds to that of the FF excitation. Hence, the chromatic aberrations of the element are dictated by the material properties in the FF spectral range. The bandwidth of the second harmonic imaging is limited by the nonlinear optical response of the metasurface. This may be broadened by engineering the metasurface or by constructing a hybrid element composed of several layers of metasurfaces,^49^ which will provide a high nonlinear optical response at different bands. The spatial resolution of the element is determined via the diffraction-limited spot,^46^ or by the Fresnel number, which was shown to be a more appropriate metric for metalenses.^50^ If we take into account the wavelength of the generated SH, both metrics are improved by a factor of 2 compared to the FF imaging. The specific HNML presented in this work possesses a rather low numerical aperture since the fabricated metasurface is small, relative to the size of the lens. The diffraction-limited spot can be improved fairly easily by fabricating a metasurface that will cover the entire lens, which will allow the full utilization of the spatial resolution the refractive lens offers. Such fabrication may require cutting the membrane to prevent wrinkles and deformability of the meta-skin, and this issue should be addressed at the metasurface design stage. Please note that, despite the small numerical aperture, the generated SH light focusing, performed by the HNML, is in agreement with the theory, as presented in Figure 4(a).

Furthermore, the metasurface can encode additional optical power to the hybrid lens. By proper design, this optical manipulation can compensate for the group delay difference in the operational band,^50^ and as a result, the overall combined optical response of the metasurface and the refractive lens can be achromatic.

In conclusion, we introduce here a new concept of hybrid nonlinear metasurface refractive elements. The nonlinear metasurface functionalizes the linear refractive element with a new capability, the ability to generate SH light and effectively refract it in a manner similar to that of the exciting illumination. We studied the concept experimentally by coating an off-the-shelf optical lens with a nonlinear plasmonic metasurface skin. The results show that our hybrid element focuses the generated SH light and can also be used for nonlinear imaging at the SH. The HNML is a sole example of how one can expand the capabilities of classical optical elements. This concept can be used also to coat refractive elements made from materials that are opaque in the converted frequency band, while transparent in the pump frequency, e.g., for harmonic generation and its refraction or also for THz generation and its refraction.

Therefore, the hybridization of nonlinear metasurfaces and standard optical components paves the way for the development and testing of a new family of nonlinear metasurface-based refractive elements and sensors.

## Experimental Methods

### Measurement Setup

In our measurement setup, the light first propagated through a broadband half-waveplate and a wire grid polarizer that enabled control over the incident wave polarization state as well as its power. Next, L1, a 200 mm focal length lens, focused the light, and the beam arrived at our HNML. After this element, light was collected by a 20 mm WD microscope objective lens (20×, 0.42 numerical aperture, infinity corrected), followed by its compatible tube lens (TL). The MO lens was placed on a linear stage with the aim of imaging the generated SH beam across different planes of the optical axis. Next, two identical 200 mm focal length lenses, L2 and L3, were set in a 4f configuration to project the light onto the detectors.

The linear transmission of the HNML was measured by illuminating it with a supercontinuum laser (SuperK COMPACT, NKT) and capturing the transmitted light by an imaging spectrometer, a cooled back-illuminated EMCCD detector (Andor Shamrock 303i, Newton 970), for the wavelength band 450–900 nm or by a near-infrared spectrometer suitable for the wavelength range of 900–1600 nm (Ocean optics, NIRQuest-512). For the latter, the light was deflected by a mirror after L2 into the fiber-coupled spectrometer that was placed at the L2 focal plane (not included in the illustration of Figure 2).

For the nonlinear measurements, we used a tunable femtosecond optical parametric oscillator pumped by a titanium sapphire laser (Chameleon OPO VIS, pulse width ∼140 fs, repetition rate 80 MHz) as the FF source with a wavelength span in the range of 1040–1580 nm. In this experimental part, we incorporated a long pass filter (LPF) after L1, close to the HNML, to prevent any stray light within the SH spectral range, which did not originate from the HNML, from propagating through the optical path. In addition, the transmitted FF power was filtered after the HNML by a short pass filter (SPF). For SH polarization resolved analysis, a broadband polarizer (A) was added before the imaging spectrometer.

During the nonlinear measurements, we utilized the dual functionality of our imaging spectrometer and examined both the spectral response and the HNML effective refraction characteristics by recording images of the SH beam.

## Supplementary Material



## Notes

Details on the fabrication process and the FDTD simulation (PDF)

## Abbreviations

HNML: hybrid nonlinear metasurface lens
SH: second harmonic
FF: fundamental frequency
SEM: scanning electron microscope
PMMA: poly­(methyl methacrylate)
DI: deionized
λ/2: half-waveplate
P: polarizer
WD: working distance
MO: microscope objective
TL: tube lens
LPF: long pass filter
SPF: short pass filter
A: analyzer
H-pol: horizontally polarized
V-pol: vertically polarized
FDTD: finite difference time domain
ITO: indium-tin-oxide
