# Supplementary material for: Hybrid Nonlinear Metasurface Refractive Lens
Source: Nano Lett. 2025 Apr 3;25(20):8103–9. doi: 10.1021/acs.nanolett.5c00178 (PMC12100703; doi:10.1021/acs.nanolett.5c00178)
Supplement: Supplementary file 1 [file nl5c00178_si_001.pdf]

Supporting information:

Hybrid nonlinear metasurface refractive lens

Sharon Karepov[1,2,*], Costantino De Angelis[3] and Tal Ellenbogen[1,2]

[1] Department of Physical Electronics, Faculty of Engineering, Tel-Aviv University, Tel-Aviv 6997801, Israel

[2] Light Matter Interaction Center, Tel Aviv University, Tel Aviv 6997801, Israel

[3] Department of Information Engineering, University of Brescia, Via Branze 38, 25123 Brescia, Italy

* sharond3@mail.tau.ac.il

Contents

Fabrication process S1

Finite Difference Time Domain (FDTD) simulation S2

Fabrication process

The concept of hybridizing metasurfaces and optical components is enabled thanks to the ability to embed the meta-atoms in an ultrathin transparent membrane that allows the transferring of the metasurface to the goal substrate. The fabrication scheme is shown in Fig. S1. The first step was

to fabricate the 1×1mm^2 metasurface, composed of V-shaped gold nanoparticles, via standard electron beam lithography (Raith 150 II). We spin coated Poly(methyl methacrylate) (PMMA) A4 (Michrochem) on an indium tin oxide (ITO) coated glass and baked it on a hotplate at 180℃ for one minute. Then, this coated substrate underwent locally selective exposure to a focused electron beam, resulting in a targeted change of the PMMA solubility properties. The exposure occurred only in the regions that were later covered by the meta-atoms metal, as shown in Fig. S1(a). Next, by performing chemical development, the PMMA that was exposed to the electron beam was removed (see Fig. S1(b)). Then, we evaporated, in a VST electron beam evaporator, 40nm of gold onto the wafer with no adhesion layer, in order to facilitate the separation of the meta-atoms from the ITO-coated glass. The undesired gold and PMMA residues were removed in an over-night liftoff in acetone. At this stage, we obtained a 1×1mm^2 metasurface on an ITO-coated glass, as depicted in Fig. S1(d).

In order to transfer this metasurface from the ITO-coated glass, we spin coated ~400nm of PMMA A4 (Michrochem) and baked the element on a hotplate at 180℃ for one minute. The PMMA serves as the meta-atom holding membrane. To provide the delicate ultrathin PMMA layer with mechanical support, a Kapton frame was taped on the PMMA layer and around the metasurface. These steps can be seen in the illustration of Fig. S1(e, f), respectively. At this point, the meta-atoms were encapsulated in the PMMA membrane but still attached to the ITO-coated glass. To separate the metasurface from the ITO-coated glass, we soaked the element in deionized water for four hours. During this time, capillary forces rise and separate the PMMA from the ITO-coated glass. This separation is illustrated in Fig. S1(g), and it enables, by a gentle tweezers grip, to lift the metasurface-PMMA meta-membrane from the glass substrate, as shown in Fig. S1(h).

The final step was to place the meta-membrane onto the convex side of the refractive lens (Thorlabs LA1252) while applying slight pressure in order to attach the meta-membrane to the refractive lens. The natural adhesion of the PMMA was sufficient to bond the meta-membrane to the lens facet. At the end of this step, which is depicted in Fig. S1(i), left, we obtained the HNML that is shown in Fig. S1(i), right.

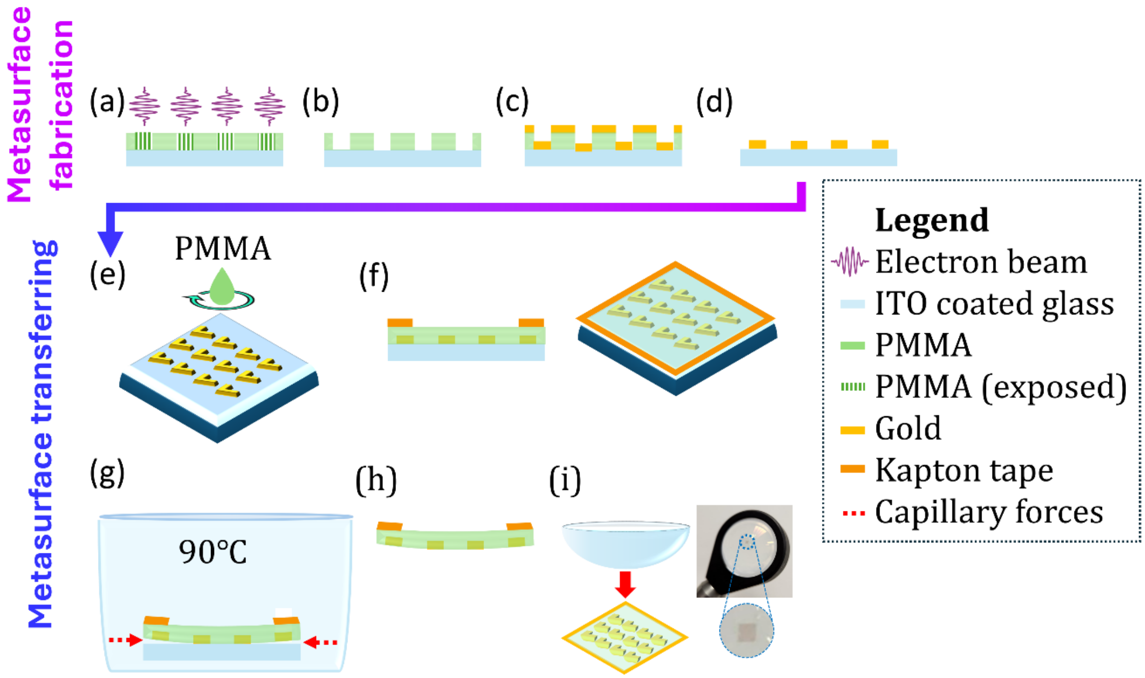

Fig. S1 HNML fabrication scheme. (a) Locally selective exposure of PMMA spin coated on an ITO-coated glass to a focused electron beam. (b) Removal of PMMA in regions that were exposed to the electron beam by chemical development. (c) Electron beam evaporation of 40nm thick gold layer. (d) Removal of undesired PMMA and gold by an overnight liftoff in acetone. (e) 400nm PMMA spin coat to create an ultrathin membrane that will hold the meta-atoms during the following fabrication steps. (f) Kapton frame tape on the PMMA around the metasurface. Left, side view; right, perspective view. (g) Element immersion in deionized water for four hours. (h) Free-standing meta-membrane after gentle peeling it from the ITO-coated glass. (i) Left, locating

the meta-membrane on the convex side of a refractive lens and applying mild pressure to bond between the PMMA membrane and the lens; Right, a picture of the resulting HNML, top, and a zoom in of the metasurface region, bottom.

Finite Difference Time Domain (FDTD) simulation conditions

Finite Difference Time Domain linear simulations were conducted in Ansys Lumerical. The illumination was defined as a Bloch plane wave propagating at normal incidence. We simulated a single unit cell using periodic boundary conditions. Considering the thickness of the lens glass is 10^4 orders of magnitude larger than the simulated illumination wavelengths, it can be considered as semi-infinite. Therefore, the unit cell included an NBK7 layer, with an infinite thickness at its bottom, in which the illumination source was located. The light propagated to a gold V-shaped meta-atom placed on top of the NBK7 layer and surrounded by a 400nm thick PMMA layer. For the optical axis, we employed perfectly matched layer boundary conditions.

We used several material models in the simulation. The model of the meta-atom Au was part of the material properties library built in Lumerical and was based on the data published by Palik[1].

Since the published optical properties of the refractive lens glass, NBK7, are limited, we used data taken from the manufacturer catalogue[2].

Lastly, the material properties of PMMA A4 were extracted from the paper of Zhnag et al.[3].

To optimize the fit parameters of the material models in the simulation, we divided the illumination spectral band into two ranges. The first spectral segment, primarily in the visible regime, spanned the wavelength range of 450-800nm, while the second segment covered the spectrum between 800 and 1550nm. This segmentation allowed us to obtain an independently optimized fit for the material coefficients in the simulation for each band.

References

1. Palik, Edward D. *Handbook of Optical Constants of Solids*. Elsevier 1998.

2. Link to the Schott lens catalogue: https://www.schott.com/shop/advanced-optics/en/Optical-Glass/SCHOTT-N-BK7/c/glass-SCHOTT%20N-BK7%C2%AE.

3. Xiaoning Zhang, Jun Qiu, Xingcan Li, Junming Zhao, and Linhua Liu, "Complex refractive indices measurements of polymers in visible and near-infrared bands," Appl. Opt. 59, 2337-2344 (2020).
